# Noradrenergic therapies for apathy in Alzheimer’s disease?

**DOI:** 10.1136/jnnp-2022-330824

**Published:** 2022-12-05

**Authors:** Masud Husain

**Affiliations:** Nuffield Dept Clinical Neurosciences and Dept Experimental Psychology, Oxford University, Oxford, UK

**Keywords:** APATHY, NEUROPSYCHIATRY

Apathy is an important neuropsychiatric syndrome across many neurodegenerative disorders. In Alzheimer’s disease (AD), where it may affect nearly half of all patients, it is associated with poor prognosis, accelerated functional decline and high caregiver burden.^1^ Symptomatic improvement of apathy might therefore have a significant impact on both patients and their families. However, there is no licensed therapy, although several studies particularly in Parkinson’s disease (PD) have pointed to a potential role for dopaminergic drugs. Dopamine may operate, at least in part, by increasing incentivisation by reward: patients with apathy might value possible actions as more rewarding when treated with such drugs.

The meta-analysis by David *et al* 2 considers a possible role for noradrenergic therapies in AD. Their analysis across eight randomized controlled trials (RCTs), which used drugs that modulate the noradrenergic system revealed a significant positive effect on apathy. However, there was only a mild positive effect on cognition assessed with screening measures across 10 RCTs. Most of the studies on apathy have used methylphenidate, including the most recent apathy in dementia methylphenidate trial (ADMET) 2 trial, a 6-month RCT of 200 patients with AD. But in addition to increasing norepinephrine levels (by blocking the norepinephrine transporter), methylphenidate also reduces reuptake of dopamine, thereby promoting higher levels of this catecholamine. It is possible, therefore, that methyphenidate’s beneficial effects might be due to a dual effect on both noradrenergic and dopaminergic mechanisms.

What might increasing norepinephrine levels do? Apathy has been conceptualised as a disorder of motivated behaviour, a key aspect of which is how people weigh up the potential reward for pursing a course of action against the effort required to obtain it.^3^ The action might involve physical effort (eg, putting out rubbish) or require cognitive effort (eg, reading a book). If the effort is judged too high for the level of reward, effort might not be invested and the patient appear to be inert. In the subjective evaluation of reward and effort, norepinephrine has been implicated in the allocation of both physical and cognitive effort.^4^

Recordings from the locus coeruleus (LC), the principal site for brain norepinephrine synthesis, demonstrate neurons whose activity correlate with both types of effort, as well as with attentional state.^4^ The LC is affected early in both AD and PD providing further reason to consider degeneration of this structure to be crucial for some of the manifestations common to both conditions, such as apathy.

Most clinicians are not familiar with prescribing methylphenidate though, which in the UK is a controlled drug. This makes its use problematic, even if it is licensed for attention deficit hyperactive disorder. Atomoxetine, a more selective norepinephrine reuptake inhibitor, is easier to prescribe but has far less of an evidence base in apathy.^2^ Further trials using this drug are therefore warranted, ideally in a direct comparison with methylphenidate. It remains a distinct possibility though that methylphenidate is more effective because it alters both incentivisation by reward (dopamine) and willingness to allocate effort (norepinephrine). Regardless, there is now definitely sufficient reason to consider treatment of apathy by repurposing existing drugs.
